# Dental Items

**Published:** 1870-12

**Authors:** 


*Dental Items.*—In the November No. of the *Canada Journal of Dental Science*, a writer recommends cotton thoroughly saturated with *os-artificiel*, which, (while soft,) is pressed between crowded teeth which have been previously separated, to retain the space already gained until the sensitiveness has in a great measure passed away, thereby saving the renewal of the wedges.

For years past we have been using for this purpose, cotton saturated with sandarach varnish, which hardens as soon as water comes in contact with it, saving the time necessary for *os-artificiel* to harden, and besides being more conveniently applied.

In the November No. of the *Dental Cosmos*, a writer recommends the use of *os-artificiel* in vulcanite work, as a protection to the joints between the blocks of gum teeth. This material has long been used for this purpose, and the suggestion is not a new one; but closely ground surfaces afford better protection than any material either pressed between the joints or put over them. In the first instance, if the *os-artificiel* is placed between the joints, a thickness of it will show; or a space corresponding in size to the quantity of the material applied over the joint be left, especially between the gum extremity of the blocks and the rim of rubber.

In the *Dental Office and Laboratory*, Dr. O. Lund, alluding to the difficulty of soldering aluminium, says: "I have never failed in making the solder adhere by using a spirit lamp, the "extra" solder, and a small soldering tool made after the pattern of a common soldering iron. The plate is first made clean by *thoroughly* scraping it, and is then merely "tinned" with the aluminium solder, scraping the excess off, and the block-tin, or any of the fusible metals will flow upon it without any trouble."

Dr. Lund suggests that "the manufacturer coat both sides of an ingot of aluminium with pure tin, united to the ingot by means of a solder composed of an alloy of the two metals." "Of course these coats must have a much less relative thickness than the ingot, each coat being, say, one thirty-second or one six-teenth the thickness of the ingot. Next roll the whole out in a mill till it is sufficiently thin for dental plates, and we have as the result an aluminium plate, covered with a thin coating of tin, ready for "striking up" and soldering, without difficulty."

He also claims another advantage by the method he has detailed; "we avoid the galvanic action that is apt to be generated by the aluminium metal and the solder or tin being brought into contact, side by side, with the membranes and fluids of the mouth."

This suggestion of Dr. Lund's is one of interest to the profession, and may lead to a method by which the difficulty in working aluminium may be greatly lessened.

